# The Hypotension Prediction Index in Free Flap Transplant in Head and Neck Surgery: Protocol of a Prospective Randomized Controlled Trial

**DOI:** 10.3390/life15030400

**Published:** 2025-03-04

**Authors:** Jakub Szrama, Agata Gradys, Amadeusz Woźniak, Zuzanna Nowak, Tomasz Bartkowiak, Ashish Lohani, Krzysztof Zwoliński, Tomasz Koszel, Krzysztof Kusza

**Affiliations:** Department of Anesthesiology, Intensive Therapy and Pain Management, Poznan University of Medical Sciences, 60-355 Poznan, Poland; agata.gradys@usk.poznan.pl (A.G.); amadeusz.wozniak@usk.poznan.pl (A.W.); zuzanna.nowak@usk.poznan.pl (Z.N.); tomasz.bartkowiak@usk.poznan.pl (T.B.); ashish.lohani@usk.poznan.pl (A.L.); krzysztof.zwolinski@usk.poznan.pl (K.Z.); tomasz.koszel@usk.poznan.pl (T.K.); krzysztof.kusza@usk.poznan.pl (K.K.)

**Keywords:** hypotension prediction index, perioperative hemodynamic management, free flap surgery

## Abstract

Introduction: Microvascular free flap surgery is a treatment method for patients with head and neck cancer requiring reconstruction surgery. Patients undergoing this complex, long-lasting surgery are prone to prolonged episodes of intraoperative hypotension, which is associated with increased incidence of postoperative mortality, morbidity, and free flap failure. A new technology recently approved, named the Hypotension Prediction Index (HPI), allows precise hemodynamic monitoring of patients under general anesthesia, with a significant reduction of intraoperative hypotension events. This study aims to assess the impact of the Hypotension Prediction Index (HPI) on the incidence and severity of intraoperative hypotension in patients undergoing free flap surgery. Methods and analysis: Eligible patients will be randomly assigned to one of two groups: Group A, receiving invasive blood pressure monitoring with standard medical therapy, or Group B, undergoing hemodynamic monitoring using the Hypotension Prediction Index (HPI) software. The primary outcome is the time-weighted average (TWA) of mean arterial pressure (MAP) < 65 mmHg. Secondary outcomes include free flap viability and perioperative complications. Ethics and dissemination: Ethics approval was obtained from the Poznan University of Medical Sciences Ethics Committee (KB-560/22; date 1 July 2022). Results will be submitted for publication in a peer-reviewed journal. Trial registration number: NCT 05738603.

## 1. Introduction

Microvascular free flap surgery is a treatment method for patients with head and neck malignancies requiring reconstructive surgery after the resection of the tumor. Intraoperative anesthetic management of patients undergoing these complex, long-lasting surgeries is one of the key elements of successful patient outcomes. There are several definitions of intraoperative hypotension (IOH), and the incidence of IOH, according to the different definitions, can range from 5–99% [1]. Growing evidence suggests that even brief episodes of intraoperative hypotension are associated with an increased risk of mortality in the perioperative period, myocardial infarction, acute renal failure, and brain ischemic incidents [2,3,4,5,6,7,8]. A retrospective analysis by Jason et al., which included 445 patients undergoing free flap surgery, showed that episodes of hypotension are a significant risk factor for flap failure [9]. Hemodynamic stability during free flap surgery is established by both fluid, inotrope, and vasopressor administration [9]. The administration of vasopressor agents raises concerns regarding their effects on the microvessels (i.e., vasoconstriction) with the risk of impaired free flap blood flow [10]. On the other hand, hypotension creates a risk for diminished flow to the anastomosed tissue. Fluid therapy may also have certain side effects. It has been shown that fluid overload may impair the viability of the free flap [11].

Hemodynamic stability is the cornerstone of anesthesia management in high-risk patients or during surgical procedures with a high risk of complications. Monitoring the cardiovascular system using invasive blood pressure-derived cardiac output measurement and integrating these parameters into perioperative hemodynamic management appears essential for delivering high-quality anesthesia. The so-called goal-directed therapy aimed at improving oxygen delivery to the tissues and reducing perioperative complications proved to be beneficial in some studies [12,13,14]. Intraoperative goal-directed fluid therapy in patients undergoing head and neck cancer surgery with free tissue transfer might help in hypovolemia management and prevent inadequate microvascular perfusion [15]. Despite using advanced hemodynamic monitoring as an APCO technique, the anesthetic treatment of hypotension remains reactive, i.e., one makes an intervention when the episode of hypotension arises. Instead of preventing the occurrence of hypotension, the attending anesthesiologist administers fluids, vasopressors, or inotropes when the drop in blood pressure is already affecting vital organs. A new technology has recently appeared on the market that determines the Hypotension Prediction Index by analyzing the arterial pressure waveform and giving a numerical value, which indicates a risk of hypotension to occur, defined as MAP below 65 mmHg for one minute [16]. The HPI sensitivity and specificity 5 min before the episode of hypotension are both 92% and, respectively, 89% and 90% 10 min before the hypotension occurrence [16]. Although the method seems promising, only a few studies have investigated the effects of the use of this technology in preventing severe hypotension and also the effects on postoperative outcomes [17,18,19,20,21,22,23,24].

This study aims to assess the impact of the Hypotension Prediction Index (HPI) on intraoperative hypotension in patients undergoing free flap surgery. Additionally, it will explore the relationship between hypotension episodes and the viability and function of free flaps. We hypothesize that the use of the HPI algorithm will reduce the time-weighted average (TWA) of intraoperative hypotension, keeping mean arterial pressure (MAP) above the 65 mmHg threshold in patients undergoing free flap transplantation

## 2. Materials and Methods

### 2.1. Study Design

This prospective, randomized, controlled trial will be conducted at the Poznan University of Medical Science Hospital. Eligible patients (see Section 2.6 for criteria) are going to be assigned to Group A, receiving standard medical therapy with invasive blood pressure monitoring, or Group B, undergoing hemodynamic monitoring using the Hypotension Prediction Index software (Acumen IQ sensor, Edwards Lifesciences, Irvine, CA, USA).

Ethics committee approval was obtained from the Poznan University of Medical Science Bioethics Committee (KB-560/22; date 1 July 2022). The trial was registered in clinical trials under the number NCT 05738603.

### 2.2. Randomization

A randomization method using sealed envelopes is performed. A sealed envelope will be opened by the recruited patient with randomization to either group A or group B. For each of the ten envelopes, five patients will be recruited to Group A and five patients to Group B.

### 2.3. Blinding

Patients recruited for the study will not be aware of the group allocation. Due to the nature of advanced hemodynamic monitoring, it is not possible to keep the anesthesiologists aware of the group allocation of the patients. However, the analysis of the data will be kept blind.

### 2.4. Duration

Patients will be randomized to the study the day before their surgical procedure. Demographic, clinical, and laboratory data will be collected prior to surgery. Hemodynamic monitoring will begin before anesthesia induction and continue until the end of anesthesia in the operating room. Postoperative screening will be conducted on the day of surgery, on the 1st and 2nd postoperative days, at the time of hospital discharge, and during the first postoperative visit. Patients will also be followed up on day 30 after randomization

The planned starting date of the study is 1 March 2025, and the planned finishing date is 30 September 2026.

### 2.5. Study Groups

Patients meeting the inclusion and exclusion criteria (defined below) will be randomly allocated to one of two study groups. Group A will receive invasive blood pressure monitoring along with standard medical therapy, while Group B will undergo hemodynamic monitoring using the Hypotension Prediction Index.

### 2.6. Patient Enrolment

#### 2.6.1. Inclusion Criteria

Patients qualified for head and neck free flap surgery and required written informed consent.

#### 2.6.2. Exclusion Criteria

The following are the exclusion criteria:

- Patients under 18 years;
- Lack of health insurance;
- Pregnancy;
- A known history of any of the following conditions:
  - congenital heart disease;
  - severe aortic and/or mitral stenosis;
  - heart failure and ejection fraction < 35%;
  - persistent atrial fibrillation and other arrhythmias impairing APCO monitoring.

### 2.7. Outcomes of the Study

#### 2.7.1. Primary Endpoint

TWA − MAP < 65 mmHg: Time weighed average of mean arterial pressure below 65 mmHg (depth of hypotension [in mmHg] below the specified MAP threshold of 65 mmHg × time [in minutes] spent below the specified MAP threshold of 65 mmHg)/total duration of the operation (in minutes).

#### 2.7.2. Secondary Endpoints

TWA − MAP < 50 mmHg.

TWA − MAP > 90 mmHg.

TWA − MAP > 100 mmHg.

30-day mortality.

Length of hospitalization.

Viability and function of free flap: NIRS tissue oximetry values (a serious free flap ischemia will be defined as StO_2_ < 30% or a drop of StO_2_ more than 20% from the baseline value), the need for reoperation due to free flap necrosis or other complications, free flap loss;

Myocardial injury evaluated by postoperative troponin levels defined as hsTnT > 65 ng/L).

Kidney injury evaluated based on postoperative serum creatinine levels (increase in sCr by > 0.3 mg/dL within 48 h; increase in sCr to 1.5 times baseline; or urine volume less than 0.5 mL/kg for 6 h).

Postoperative delirium evaluated by the NuDESC: Nursing Delirium Screening Scale.

The amount of fluids used intraoperatively.

The total amount of vasopressors used intraoperatively.

The TWA of MAP will be calculated using the Acumen Analytics software (version 3.0.2) by Edwards Lifesciences. The mathematical equation for the TWA of MAP below 65 mmHg is presented as follows:

$$\mathrm{TWA}\;\mathrm{MAP}\;<\;65\;\mathrm{mmHg}=\frac{AUC\;of\;MAP<65\;mmHg\;(mmHg\;\times \;min)}{total\;duration\;of\;surgery\;in\;minutes}$$

where AUC is the area under the curve and the total area under the MAP of 65 mmHg.

### 2.8. Patient and Public Involvement

Patients or the public were not involved in the design, conduct, reporting, or dissemination plans of our research.

## 3. Study Protocol

### 3.1. Interventions

All patients will undergo general anesthesia with standard drug regimens. In addition to routine monitoring, an arterial line will be inserted into the radial artery (in cases of technical/anatomical problems, a femoral artery catheter will be placed) prior to anesthesia induction for all patients.

#### 3.1.1. Group A: Standard Therapy

Patients in the standard therapy group will undergo invasive blood pressure monitoring and receive standard medical therapy. Decisions regarding the management of hypotension, including the administration of fluids, inotropes, and vasopressors, will be made by the anesthesia team.

#### 3.1.2. Group B: HPI Monitoring

**Figure 1:**
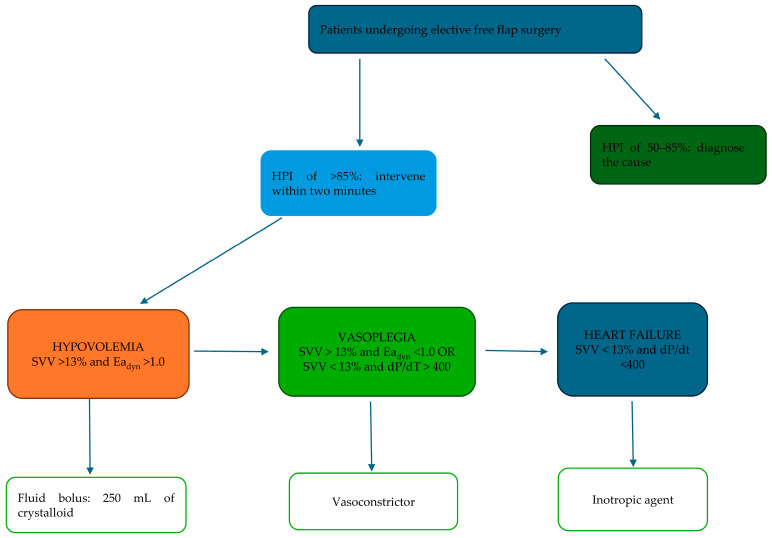
HPI hemodynamic algorithm reprinted with permission from ref. [25]. (COPYRIGHT © 2023 Lorente, Ripollés-Melchor, Jiménez, Becerra, Mojarro, Fernández-Valdes-Bango, Fuentes, Moreno, Agudelo, Villar-Pellit de la Vega, Ruiz-Escobar, Cortés, Venturoli, Quintero, Acedo, Abad-Motos, Gómez, AbadGurumeta and Monge García) (the algorithm adopted from the pReDictH trial [25]). Abbreviations: dP/dt, the rate of pressure change with time during isovolumic contraction of the cardiac ventricles; Ea_dyn_, dynamic arterial elastance; SVV, stroke volume variation.

In the HPI group, the Acumen IQ sensor will be used together with the HPI hemodynamic algorithm. Hemodynamic management will follow the HPI guidelines and the algorithm shown in Figure 1, addressing hypovolemia, decreased contractility, and vasoplegia. When the HPI value exceeds 85, an alert will prompt the clinician to intervene and take appropriate action to prevent a drop in mean arterial pressure below 65 mmHg.

However, due to the fact of acquiring invasive blood pressure data and analysis of the data by the Acumen Analytics software (Edwards Lifesciences), patients in the standard therapy group are going to be monitored with the Hemosphere platform and the Acumen IQ sensor, but the monitor will be blinded to the attending anesthesiologist.

Hemodynamic management related to the trial will conclude at the end of the surgery. Patients will then be transferred to the postoperative unit or the intensive care unit based on clinical indications

### 3.2. Measurements

#### 3.2.1. Preoperative Data

Basic demographics, including age and ASA physical status, will be collected and analyzed. The history of comorbidities, including heart, lung, and kidney diseases, will also be documented. Preoperative results of all basic, routine laboratory tests, including troponins and NTproBNP, will be recorded.

#### 3.2.2. Perioperative Data

Anesthetic data will include the total amount of opioids, intravenous anesthetics, and volatile anesthetics, expressed as MAC hours. Additional parameters to be recorded include BIS values, esophageal temperature, urine output, blood loss, estimated evaporative fluid loss, and pulmonary and hemodynamic parameters such as heart rate (HR) and arterial blood pressure (ABP). The total amount of catecholamines, including noradrenaline, dobutamine, and intraoperative fluids) will also be documented, along with calculations of intraoperative and postoperative fluid balance.

Blood pressure measurements will include data from standard invasive blood pressure monitoring in Group A and from the hemodynamic monitoring platform analyzed by the Acumen IQ transducer in Group B. Parameters recorded intraoperatively will include heart rate (HR), systolic, mean, and diastolic blood pressure, cardiac output, cardiac index, stroke volume, stroke volume index, stroke volume variation, pulse pressure variation, systemic vascular resistance, systemic vascular resistance index, and dynamic arterial elastance.

During the free flap transplantation procedure, a ForeSight tissue oximetry sensor will be attached to the transplanted tissue in order to measure tissue oximetry StO_2_.

#### 3.2.3. Follow-Up

Patients will be screened in the postoperative period for specific organ injury. On the day of surgery, 1st and 2nd postoperative day, troponin, NTproBNP, and creatinine levels will be assessed. Similarly, postoperative delirium screening with the NuDESC scale will be performed on the day of surgery, 1st and 2nd postoperative day, on the day of hospital discharge, and on the following postoperative visits in the ambulatory setting. Patients will be evaluated for any other complications with a major impact on free flap failure by the decrease in near-infrared spectroscopy tissue oximetry, need for reoperation due to free flap failure, and free flap loss. Secondary outcomes, including hospital length of stay, will be evaluated in other studies.

## 4. Statistical Analysis

### 4.1. Sample Size Calculation

An analysis of the retrospective data from patients undergoing major noncardiac surgery showed a mean time weighed an average of mean arterial pressure below 65 mmHg of 1.18 ± 2.51 mmHg with the use of the APCO hemodynamic system and 0.20 ± 0.32 mmHg with hypotension prediction index system [26]. Based on these values, the predicted sample size was 103 patients per group, with the consideration of 80% power and a type 1 error of 5% (*p* < 0.05) calculated using an online platform [27].

### 4.2. Study Populations

Adult patients undergoing free flap transplant surgery due to head and neck cancer fulfilling the inclusion and exclusion criteria.

### 4.3. Study Groups

Group A: Invasive blood pressure monitoring + standard medical therapy.

Group B: Invasive blood pressure monitoring + Hypotension Prediction Index Hemodynamic Monitoring.

### 4.4. Statistical Analysis

The normality of the data distribution will be evaluated using the D’Agostino–Pearson test. Continuous variables will be expressed as the mean ± standard deviation for normally distributed data or as the median (interquartile range) for non-normally distributed data. Categorical variables will be reported as frequencies (percentages).

Individual analyses will be performed to assess the relationship between predictor variables and the outcome. The chi-square test or Fisher’s exact test will be used to compare categorical variables between groups, the Student’s *t*-test or Wilcoxon test will be applied to assess differences in continuous outcomes, and binary logistic regression will be conducted to evaluate the impact of multiple factors on a binary outcome.

A *p* < 0.05 will be considered statistically significant. However, the Bonferroni correction will be used to reduce the risk of Type I errors. All statistical analyses will be performed using the MedCalc^®^ statistical software (version 20.115; MedCalc Software Ltd., Ostend, Belgium).

## 5. Study Phases

### 5.1. Enrolment

**Figure 2:**
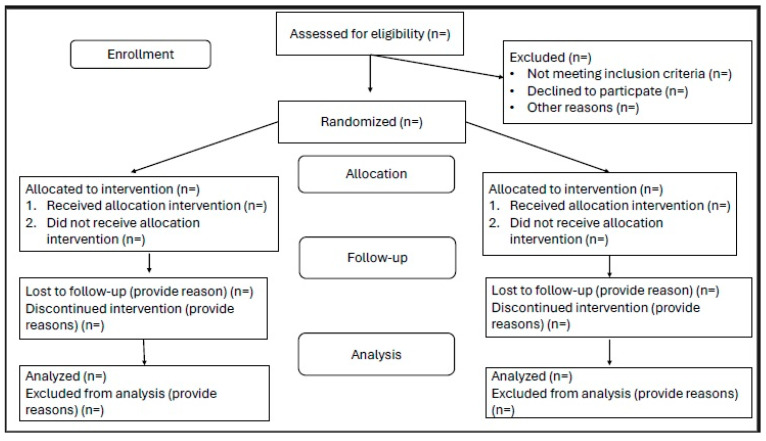
A Consolidated Standards of Reporting Trials (CONSORT) flowchart of the trial.

All patients scheduled for free flap transplantation due to head and neck cancer will be screened to determine their eligibility based on the study inclusion and exclusion criteria. Participants will provide informed consent on the day before surgery. Demographic information and data regarding the comorbidities are going to be documented before randomization. The randomization will be conducted the day prior to surgery using a sealed envelope method. Patients will not be aware of the group allocation. Although the anesthesiology personnel will not be blinded to group assignments, the analysis of the data will be performed in a blinded manner. All the anesthesiologists taking part in the study are going to have guidance in advanced perioperative hemodynamic management and adherence to the study protocol outlined in Figure 1. A Consolidated Standards of Reporting Trials (CONSORT) flowchart for this trial is provided in Figure 2.

### 5.2. Treatment Phase

Patients in both groups are going to have general anesthesia with basic monitoring, a peripheral and central intravenous line, and an indwelling radial arterial catheter. The choice of drugs for anesthesia induction and neuromuscular blockade will be at the discretion of the attending anesthesiologist. Anesthesia will be maintained with sevoflurane, targeting a BIS range of 40–60.

Patients in Group A will receive invasive blood pressure monitoring and hemodynamic monitoring with the Acumen IQ sensor; however, the Hemosphere platform will be blinded to the attending anesthesiologist, providing no advanced hemodynamic data. Patients in Group B will receive hemodynamic monitoring using the hypotension prediction index algorithm. Hemodynamic monitoring in both groups will commence prior to anesthesia induction.

Standard maintenance fluids will be administered during the intraoperative period. Red blood cells are going to be transfused when the hemoglobin levels drop below the 8 g/dL threshold.

In Group A, the anesthesiologists are going to maintain mean arterial pressure above 65 mmHg. The hemodynamic management is going to be based on data derived from invasive arterial pressure measurement with the administration of fluids, inotropes, and vasopressors based on the decision of the attending anesthesiologist.

In Group B, the anesthesiologist is going to maintain the mean arterial pressure above 65 mmHg with the use of the Hypotension Prediction Index algorithm. The Hypotension Prediction Index algorithm is presented in Figure 1. According to the algorithm, there are three basic elements responsible for episodes of hypotension: hypovolemia, decreased contractility, and vasoplegia. If the HPI value reaches 85 secondary screen parameters, they need to be analyzed in order to prevent the occurrence of hypotension. According to the algorithm, fluid should be administered if the SVV is above 13% and the Ea_dyn_ is above. An inotropic agent should be infused when the SVV value is below 13%, and dP/dt is below 400, which indicates decreased contractility.

A vasoconstrictor agent should be infused if the SVV value is above 13%, but the Ea_dyn_ is below 1, or when the SVV is below 13%, and dP/dt is above 400.

The Hemosphere platform is going to document the hemodynamic parameters every 20 s. The data will be downloaded afterward for analysis using the Acumen Analytics software (Edwards Lifesciences).

During the free flap transplantation procedure, a ForeSight tissue oximetry sensor will be attached to the transplanted tissue to measure StO_2_ tissue oximetry.

### 5.3. Daily Assessment

Basic laboratory tests are going to be analyzed in the perioperative period on the day of surgery and the 1st and 2nd postoperative days. Preoperatively, patients will be screened for delirium using the NuDESC screening tool. Similar tests will be performed on the day of surgery, 1st and 2nd postoperative days, and on the first visit after hospital discharge.

During the postoperative period, the tissue oximetry values will be measured continuously with suspicion of free transplant ischemia in the situation of StO_2_ values < 30% or a drop >20% from the baseline for one hour [27,28,29].

### 5.4. Data Management

All patient data will be handled anonymously in an electronic database made specially for the study.

### 5.5. Follow-Up

Follow-ups will be conducted 30 days after randomization using a follow-up phone call and on the first ambulatory visit of the patients after hospital discharge. In case we are unable to reach the patient or the next-of-kin, we use medical records to obtain the needed information. On day 30, survival will be assessed, and delirium screening will be performed on the first postoperative ambulatory visit. An overview of the outcome assessments is provided in Table 1.

## 6. Discussion

The goal of the study is to determine whether advanced hemodynamic monitoring with the use of the HPI algorithm will reduce the rate of hypotension in patients undergoing free flap transfer. Growing evidence suggests that even brief episodes of intraoperative hypotension are associated with an increased risk of mortality in the perioperative period, myocardial infarction, acute renal failure, and brain ischemic incidents [3,4,5,6,7]. The long surgical time during free flap transfer surgeries and the fear of using vasopressors due to the impairment of perfusion of the flap stand as risk factors for hypotension during these surgeries. Hypotension itself is also considered a risk factor for flap surgery failure [8].

Advanced hemodynamic monitoring during anesthesia allows precise titration of vasopressors and fluid therapy, as fluid overload is also considered a risk factor for free flap surgery failure [10]. In our institution, the use of a Flotrac transducer, which allows arterial waveform analysis with calculation of cardiac output, is a standard therapy for major surgeries. Despite using this type of monitoring, we performed an analysis of intraoperative data of 61 patients undergoing major abdominal surgery (unpublished data) and found that 87% of patients experienced at least one episode of hypotension <65 mmHg. The mean duration of such hypotensive event was 5 min (mean ± SD 5 ± 7 min), and the total duration of hypotension was 14.4% of surgical time. Median TWA (time-weighted average) intraoperative hypotension below 65 mm Hg was 0.31 mmHg.

The HPI algorithm allows for the proactive and preventive management of hypotension. By analyzing more than 20 features of arterial pressure waveform, the software gives a numerical value of the risk of a hypotensive event. When the value exceeds 85, there is a risk of hypotension <65 mmHg to occur in a few minutes. At the moment, there are no clinical studies that evaluate the benefits of using this type of monitoring in free flap surgeries. The results of previous studies analyzing the introduction of HPI monitoring focus mainly on the reduction of hypotensive episodes. So far, few studies proved a significant reduction in the number and duration of hypotension < 65 mmHg with the usage of the HPI algorithm [17,18,19,20,21,22,23,24]. In our study, we would like to put a step forward and analyze if the predicted decrease in the episodes of hypotension is going to affect the rate of postoperative complications such as myocardial infarction, renal failure episodes of delirium, and free flap survival.

## Figures and Tables

**Table 1:** Schedule of enrolment, interventions, and assessments. ** Specific time points listed.

	Enrollment	Study Period			
Timepoint **	D_−1_	Surgical Day (d_0_)	Daily Follow-Up (d_0 TO_ d_hd_)	Hospital Discharge (d_hd_)	Follow-Up (d_30_)
Enrollment:					
Eligibility screen	✗				
Written informed consent	✗				
Written and oral project explanation	✗				
Collection of patient demographic and comorbidity data	✗				
Allocation		✗			
Interventions:					
Group A		✗			
Group B (HPI)		✗			
ASSESSMENTS:					
Primary outcomes		✗			
Secondary outcomes		✗	✗	✗	✗

## Data Availability

The original contributions presented in the study are included in the article; further inquiries can be directed to the corresponding author.
